# Virgin Coconut Oil Attenuates Diabetic Kidney Disease via Gut Microbiota‐Metabolism‐Inflammation Axis Modulation in Type 2 Diabetic Mice

**DOI:** 10.1002/fsn3.71973

**Published:** 2026-06-11

**Authors:** Keke Shao, Yun Cao, Ruiqi Gao, Xueyun Dong, Xuehui Liu, Hao Xu, Yunhan Xie, Linlin Xu, Jiayuan He, Min Chen, Leilei Zhang, Asmaa Ali, Liang Wu, Pingping Wang

**Affiliations:** ^1^ Department of Laboratory Medicine The Yancheng Clinical College of Xuzhou Medical University, The First People's Hospital of Yancheng Yancheng China; ^2^ Department of Laboratory Medicine Taizhou Second People's Hospital Taizhou China; ^3^ Department of Laboratory Medicine, School of Medicine Jiangsu University Zhenjiang China; ^4^ Health Testing Center Zhenjiang Center for Disease Control and Prevention Zhenjiang China; ^5^ Public Experiment and Service Center Jiangsu University Zhenjiang China; ^6^ Department of Pulmonary Medicine Abbassia Chest Hospital, EMOH Cairo Egypt

**Keywords:** diabetic kidney disease, gut microbiota, inflammation, metabolomics, oxidative stress, virgin coconut oil

## Abstract

Diabetic kidney disease (DKD) poses a significant global health challenge, necessitating novel therapeutic interventions. Virgin coconut oil (CO), rich in medium‐chain fatty acids (MCFAs), has emerging metabolic benefits, but its renoprotective potential and mechanistic basis remain unexplored. This study aimed to investigate the therapeutic effects of CO on DKD and elucidate its underlying molecular mechanisms through a multimodal approach. Network pharmacology and molecular docking were employed to predict CO's bioactive targets and pathways. Experimental validation was performed in a high‐fat diet/streptozotocin‐induced type 2 diabetic mouse model, with CO administered for 12 weeks. Systemic metabolic parameters (glucose, BUN, Scr, lipid profiles) and renal function were evaluated. Renal histopathology (H&E, Masson staining), inflammatory markers (TNF‐α, IL‐6), oxidative stress indicators (GSH‐Px, SOD, MDA), and fibrosis markers (TGF‐β, Collagen IV) were quantified. Gut microbiota composition (16S rRNA sequencing) and serum metabolomic profiling (LC–MS) were analyzed to identify systemic mechanisms. Computational analysis identified octanoic acid and decanoic acid of MCFAs as principal bioactive components targeting PPARα/γ and IL‐1β, modulating PPAR signaling and oxidative phosphorylation. In vivo, CO significantly ameliorated hyperglycemia, reduced uremic toxins, and improved lipid metabolism. Renal benefits included attenuated inflammation, restored redox balance, and suppressed fibrosis. Gut microbiota restructuring revealed decreased pro‐inflammatory *Enterococcus* and enriched probiotic *Lactobacillus*. Metabolomics identified CO‐mediated regulation of arachidonic acid metabolism and riboflavin pathways. Our findings demonstrate that CO exerts comprehensive renoprotection through a novel “gut microbiota‐metabolism‐inflammation” axis, suggesting its potential as a dietary intervention for DKD. These mechanistic insights warrant further clinical investigation of CO's therapeutic applications in diabetic complications.

## Graphical abstract

This study elucidates the multi‐targeted mechanisms of Virgin Coconut Oil (CO) as a dietary intervention for ameliorating Diabetic Kidney Disease (DKD). High‐purity medium‐chain fatty acids (MCFAs, primarily octanoic acid [C8:0] and decanoic acid [C10:0]) in CO significantly remodel the gut microbiota by enriching short‐chain fatty acid (SCFA)‐producing beneficial bacteria (e.g., *Bacteroides_H* and *Lactobacillus*) and suppressing pro‐inflammatory pathogens (e.g., *Enterococcus* and *Odoribacter*). This microbial shift enhances gut barrier integrity and reduces endotoxin (LPS) translocation into the systemic circulation. Metabolically, CO promotes fatty acid β‐oxidation, modulates arachidonic acid metabolic modulatory effects, and effectively inhibits inflammatory signaling via the activation of PPARα and PPARγ receptors. Through this comprehensive modulation of the “gut microbiota‐metabolism‐inflammation axis,” CO significantly alleviates local renal oxidative stress, inflammatory responses, and tissue fibrosis. Ultimately, this leads to the amelioration of DKD symptoms, including improved fasting blood glucose, lipid profiles, and renal function markers (BUN, Scr), providing robust scientific evidence for utilizing natural functional oils in the systemic mechanisms of DKD management.

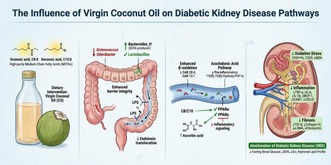

## 1 Introduction

Diabetes is characterized by persistent hyperglycemia and systemic metabolic disorders driven by intertwined insulin resistance and chronic low‐grade inflammation (Dabravolski et al. 2024; Grover et al. 2021; Zhao et al. 2023). Decreased peripheral insulin sensitivity is further exacerbated by abnormal adipokine secretion, endoplasmic reticulum stress, and pancreatic β‐cell apoptosis, forming a vicious cycle of metabolic dysfunction (Lee and Lee 2022; Moon and Jung 2022; Niranjan et al. 2023; Vesković et al. 2023; Yan 2024). Furthermore, this systemic inflammation is significantly amplified by adipose macrophage infiltration (Parisi et al. 2021; Zhao et al. 2023) and intestinal flora dysbiosis (Kang et al. 2023; Khan et al. 2021; Sultan et al. 2021), where dietary factors can trigger endotoxemia and TLR4/NF‐κB pathway activation (Lv et al. 2024; Yao et al. 2022).

Among diabetic complications, diabetic kidney disease (DKD) poses a severe threat, typically presenting with declining glomerular filtration rate and progressing to end‐stage renal disease (Joumaa et al. 2025; Młynarska et al. 2024; Yao et al. 2022). Its pathogenesis is multi‐factorial, heavily involving inflammation, fibrosis, and heightened oxidative stress driven by aberrant glucose metabolism, such as the activation of the polyol pathway (Mohandes et al. 2023).

Virgin coconut oil (CO), a functional oil rich in natural antioxidants and medium‐chain fatty acids (MCFAs)—primarily octanoic acid (C8) and decanoic acid (C10)—has emerged as a potent metabolic modulator (Ibrahim and El‐Sayed 2021; Nguyen et al. 2015; Takeishi et al. 2021). CO exhibits notable advantages in regulating lipid metabolism and improving insulin resistance (Castro et al. 2023). In the context of DKD, CO offers dual protective potential: its MCFAs reduce lipid accumulation and oxidative stress through rapid energy metabolism, while its polyphenols effectively scavenge free radicals and inhibit inflammatory signaling (Labarthe et al. 2008).

However, the specific renoprotective mechanisms of CO, particularly regarding how different MCFAs synergize and modulate host metabolism via the gut microbiota, remain largely unexplored. To address this gap, we evaluated the therapeutic efficacy of CO in a high‐fat diet/streptozotocin (STZ)‐induced type 2 diabetic mouse model. By integrating 16S rRNA sequencing and non‐targeted metabolomics, we aimed to elucidate the underlying “intestinal flora‐metabolism‐inflammation” axis. Furthermore, network pharmacology and molecular docking were employed to predict target pathways, providing a comprehensive theoretical basis for utilizing CO as a dietary intervention for DKD.

## 2 Materials and Methods

### 2.1 Analysis of Potential Targets and Pathways of Octanoic Acid and Decanoic Acid Using Network Pharmacology and Molecular Docking Techniques

In this study, all CO samples (provided by Yulin Tershan Instrument Technology Co. Ltd., Guangxi, China) were rich in octanoic acid and decanoic acid, with contents of 57.8% and 42.2%, respectively. The structures of octanoic acid and decanoic acid were retrieved from the PubChem database (https://pubchem.ncbi.nlm.nih.gov/) and imported into the CTD database (https://ctdbase.org/), SwissTarget Prediction database (http://www.swisstargetprediction.ch/), and PharmMapper database (https://lilab‐ecust.cn/) to predict drug targets. Using “Diabetes mellitus type 2” as the keyword, relevant disease targets were retrieved from the OMIM database (http://omim.org/), GeneCards database (https://www.genecards.org/), and TTD database (https://db.idrblab.net/ttd/). After merging and deduplicating target data, the relevant disease targets of T2DM were obtained. The targets of octanoic acid and decanoic acid and T2DM disease targets were used to construct “octanoic acid/decanoic acid‐target‐disease” and “Protein–Protein‐Interaction (PPI)” networks using Cytoscape software (Shannon et al. 2003). Subsequently, the core target results were imported into the DAVID database (https://david.ncifcrf.gov/) for enrichment analysis, with *p* < 0.05 as the significance threshold. GO and KEGG pathway enrichment analyses were performed using R software packages (Yu et al. 2012).

Octanoic acid and decanoic acid were selected for molecular docking with their corresponding targets. The 2D structures of octanoic acid and decanoic acid were downloaded from the PubChem database, and the active components were converted to 3D structures using Chem3D software. Key target proteins, including PPARG (peroxisome proliferator‐activated receptor gamma), PPARA (peroxisome proliferator‐activated receptor alpha), CRP (C‐reactive protein), IL1β (interleukin 1 beta), and CYP19A1 (cytochrome P450 family 19 subfamily A member 1), were downloaded in PDB format from the PDB database (https://www.rcsb.org/) and processed to remove water molecules and ligands using PyMOL software. Autodock Tools 1.5.7 was used to optimize the active components and key target proteins, which were then converted into “pdbqt” files. Autodock Vina was employed to visualize the precise binding structures between targets and small‐molecule ligands, and PyMOL View software was used for result visualization, verifying the interaction efficiency and binding affinity between octanoic acid/decanoic acid and the hypothesized targets (Seeliger and de Groot 2010).

### 2.2 Establishment of T2DM Mouse Model and Experimental Grouping

In this study, 8‐week‐old healthy male ICR mice (weight 26–32 g, provided by Jiangsu Wukong Biotechnology Co. Ltd.) were used. After 1 week of acclimatization in a standard specific pathogen‐free (SPF) environment, mice were randomly divided into four groups (*n* = 6 per group): normal control group (NC group, standard chow), T2DM model group (T2DM group), low‐dose virgin coconut oil group (COL, 10 mL/kg), and high‐dose virgin coconut oil group (COH, 20 mL/kg). Virgin coconut oil was supplied by Guangxi Yulin Huasheng Special Dietary Food Technology Co. Ltd., and high‐performance liquid chromatography (HPLC) analysis confirmed that its medium‐chain fatty acid content exceeded 98% (octanoic acid > 57% and decanoic acid > 42%).

The T2DM model was established according to the method described by Yan et al. Briefly, mice were fed a high‐fat diet (60% fat energy) for 4 weeks, followed by intraperitoneal injection of streptozotocin (STZ, 30 mg/kg dissolved in 0.1 M citrate buffer, pH 4.5) for 3 consecutive days. One week after STZ injection, fasting blood glucose (FBG) was measured, and mice with FBG > 16.7 mmol/L were considered successfully modeled. Subsequently, the T2DM, COL, and COH groups continued on the high‐fat diet. The COL and COH groups received daily intragastric administration of virgin coconut oil at doses of 10 and 20 mL/kg, respectively. These dosages were selected based on previous studies demonstrating the metabolic efficacy of high‐dose coconut oil in murine models (Yeap et al. 2015; Zhang, Wang, et al. 2023), and no adverse effects (e.g., aspiration or distress) were observed during the administration period. The NC and T2DM groups received an equal volume of normal saline via gavage. The intervention lasted until the end of week 12. At the end of the experimental period, mice were euthanized via intraperitoneal injection of sodium pentobarbital (150 mg/kg; Sigma‐Aldrich, St. Louis, MO, USA), in strict accordance with the AVMA Guidelines for the Euthanasia of Animals. Subsequently, serum, colon contents, kidney tissue, and colon tissue were collected immediately.

### 2.3 Detection of Serum Biochemical Indicators and Renal Inflammatory Factors in Mice

Serum levels of fasting blood glucose (FBG), serum creatinine (Scr), blood urea nitrogen (BUN), triglycerides (TG), total cholesterol (TC), and renal tissue concentrations of glutathione peroxidase (GSH‐Px), superoxide dismutase (SOD), and malondialdehyde (MDA) were measured using commercial kits (Nanjing Jiancheng Bioengineering Institute, Nanjing, China). Mice were fasted for 6 h before serum collection for glucose and lipid profile analysis. Kidney tissues were rapidly excised, rinsed with PBS buffer, and 100 mg of renal cortex was homogenized in pre‐cooled PBS buffer at a 1:9 weight‐to‐volume ratio. The homogenate was centrifuged, and the supernatant was collected for oxidative stress factor detection.

For RNA extraction and qRT‐PCR assay (Primer sequence in Table S1), renal tissues were thoroughly washed with sterile normal saline. All reagents were purchased from Nanjing Vazyme Biotech Co. Ltd. (Nanjing, China). Briefly, 500 μL of FreeZol Reagent was added to renal tissue samples for homogenization to extract total RNA. The concentration and purity of extracted RNA were determined using an ultramicro spectrophotometer (BioTeke Corporation, Wuxi, China). cDNA was synthesized using the HiScript III RT SuperMix for qPCR (+gDNA wiper) kit, and qPCR was performed using the ChamQ Universal SYBR qPCR Master Mix kit in a total reaction volume of 20 μL. The PCR amplification conditions were as follows: initial denaturation at 95°C for 5 min, followed by 40 cycles of denaturation at 95°C for 10 s, annealing at 60°C for 20 s, and extension at 72°C for 20 s. The relative mRNA expression levels of target genes were calculated using the 2^−ΔΔCT^ method with β‐actin as the housekeeping gene.

### 2.4 HE Staining and Masson Staining of Mouse Kidney Tissues

Mouse kidney tissues were fixed in 4% paraformaldehyde at 4°C, embedded in paraffin, and sectioned for Hematoxylin and Eosin (HE) staining. Renal histological structure was observed under a light microscope. For Masson staining, sections were stained with Weigert's hematoxylin solution for 10 min, thoroughly washed, and then stained with Ponceau acid fuchsin solution for another 10 min. Subsequently, sections were treated with 1% phosphomolybdic acid aqueous solution for 5 min, followed by staining with aniline blue solution for 5 min. Samples were rinsed with 0.2% acetic acid aqueous solution, dehydrated with graded alcohol, cleared with xylene, and mounted with neutral balsam. Renal fibrosis was observed under a microscope.

### 2.5 Gut Microbiota Analysis in Mice

The composition and diversity of murine intestinal microbiota were assessed through 16S rRNA gene sequencing performed by Ekemo Tech Group Co. Ltd. (Shenzhen, China). Following aseptic collection of fecal samples or intestinal luminal contents in sterilized conditions, specimens were immediately flash‐frozen in liquid nitrogen and maintained at −80°C prior to processing. Total microbial DNA was extracted with quality control measures to guarantee concentrations exceeding 20 ng/μL, followed by amplification of the V3‐V4 hypervariable regions using the primer pair 341F/806R. Sequencing libraries were prepared and subjected to paired‐end sequencing (2 × 300 bp) on the Illumina NovaSeq platform, with stringent quality control ensuring acquisition of ≥ 50,000 high‐quality filtered reads per biological replicate. Subsequent bioinformatics analyses incorporated sequence quality filtering, operational taxonomic unit (OTU) clustering at a 97% similarity threshold, taxonomic classification against established microbial databases, and comprehensive α/β‐diversity evaluations. The analytical outputs encompassed microbial diversity metrics presented in graphical formats along with detailed taxonomic composition profiles at various phylogenetic levels.

### 2.6 Serum Untargeted Metabolomics and Fecal Short‐Chain Fatty Acid Analysis in Mice

Serum untargeted metabolomic profiling was conducted by Ekemo Tech Group Co. Ltd. (Shenzhen, China) using ultra‐performance liquid chromatography–tandem mass spectrometry (UPLC‐MS/MS). Following protein precipitation with methanol and supernatant collection, metabolites were separated on an HSS T3 column (100 × 2.1 mm, 1.8 μm) with gradient elution using mobile phases A (0.1% formic acid in water) and B (0.1% formic acid in acetonitrile) at a flow rate of 0.4 mL/min. Mass spectrometric detection was performed with electrospray ionization (ESI) in both positive and negative ion modes, scanning *m*/*z* 50–1200, with tandem MS/MS for structural confirmation. Raw data were processed using XCMS and MS‐DIAL software, with metabolite identification through HMDB, METLIN, and KEGG databases for pathway enrichment analysis.

Quantitative determination of fecal short‐chain fatty acids (acetate, propionate, butyrate) was performed at the Zhenjiang Center for Disease Control and Prevention via UPLC‐MS/MS. Lyophilized fecal samples were extracted with phosphate buffer (pH 2.0), centrifuged, and filtered prior to chromatographic separation on an ACQUITY UPLC BEH C18 column (2.1 × 100 mm, 1.7 μm) using 0.1% formic acid in water and methanol as mobile phases in gradient mode. Multiple reaction monitoring (MRM) was employed for mass spectrometric quantification, ensuring high sensitivity and specificity for target analytes.

### 2.7 Statistical Analysis

All experimental data were analyzed using SPSS (v26.0). Normally distributed variables were expressed as mean ± standard deviation (mean ± SD). Untargeted metabolomics data were subjected to multivariate statistical analyses, including principal component analysis (PCA) and orthogonal partial least squares‐discriminant analysis (OPLS‐DA), to assess intergroup differences. Significant differential metabolites were identified based on a variable importance in projection (VIP) threshold > 1.0 and a two‐tailed *t*‐test (*p* < 0.05). For fecal short‐chain fatty acid quantification, one‐way analysis of variance (ANOVA) was performed for multi‐group comparisons, followed by Tukey's post hoc test for pairwise assessments. Statistical significance was defined as *p* < 0.05, and all graphical representations were generated using GraphPad Prism 9.0.

## 3 Results

### 3.1 Network Pharmacology Results

**FIGURE 1:**
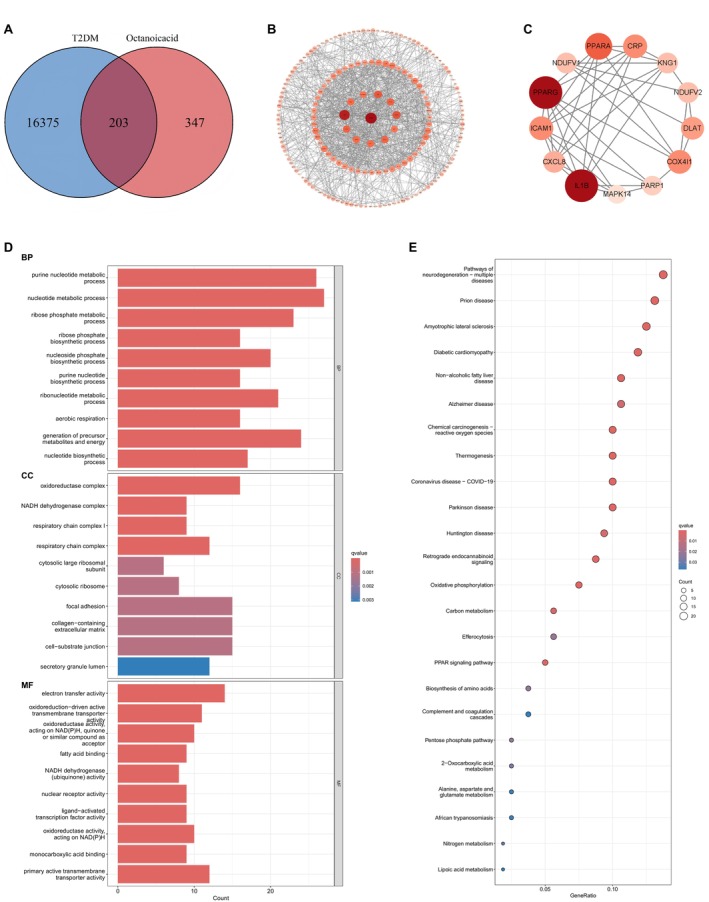
Network pharmacology analysis of octanoic acid. (A) Venn diagram illustrating overlapping targets between octanoic acid and type 2 diabetes mellitus (T2DM); (B, C) Protein–protein interaction (PPI) network of “octanoic acid‐shared targets‐T2DM”; (D) Gene Ontology (GO) functional enrichment analysis of potential therapeutic targets of octanoic acid in T2DM treatment; (E) Kyoto Encyclopedia of Genes and Genomes (KEGG) pathway enrichment analysis revealing key signaling mechanisms associated with octanoic acid's anti‐T2DM effects. This integrated approach systematically characterizes the multi‐target pharmacological profile of octanoic acid, highlighting its potential regulatory roles in biological processes and disease‐related pathways underlying T2DM pathogenesis. The significance threshold for enrichment analysis was set at *p* < 0.05.

**FIGURE 2:**
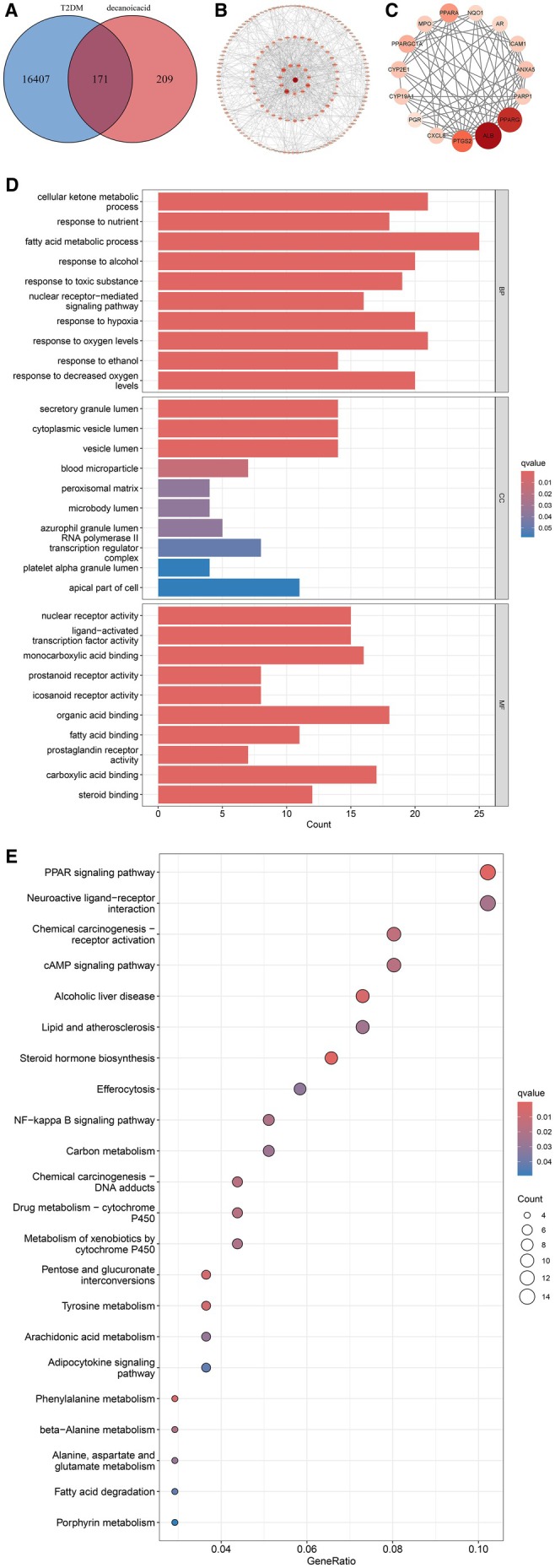
Network pharmacology analysis of decanoic acid. (A) Venn diagram demonstrating the overlapping targets between decanoic acid and T2DM; (B, C) PPI network of “decanoic acid‐shared targets‐T2DM”; (D) GO functional enrichment analysis of therapeutic targets underlying decanoic acid's anti‐T2DM effects; (E) KEGG pathway enrichment analysis identifying key signaling pathways modulated by decanoic acid. This comprehensive analysis elucidates the multi‐target mechanisms of decanoic acid in T2DM intervention, revealing its involvement in crucial biological functions and disease‐associated signaling cascades. The significance threshold for enrichment analysis was set at *p* < 0.05.

A total of 203 and 171 proteins/genes were identified as potential therapeutic targets for T2DM treated with octanoic acid and decanoic acid, respectively. The protein–protein interaction (PPI) network for octanoic acid comprised 185 nodes and 857 edges (Figure 1A). Analysis of this network revealed key hub targets, including KNG1, CXCL8, ICAM1, PPARA, MAPK14, PPARG, IL1B, PARP1, NDUFV2, CRP, DLAT, NDUFV1, and COX4I1 (Figure 1B,C). Similarly, the decanoic acid PPI network consisted of 157 nodes and 723 edges (Figure 2A), centered around core targets such as NQO1, CYP19A1, PPARG, AR, PTGS2, PPARGC1A, ANXA5, CXCL8, ALB, PGR, PO, ICAM1, CYP2E1, PPARA, and PARP1 (Figure 2B,C).

Functional Enrichment Analysis of T2DM Targets To elucidate the relevant therapeutic mechanisms of these medium‐chain fatty acids in T2DM, GO and KEGG enrichment analyses were performed using the DAVID database.

For octanoic acid, relevant biological processes (BP) were predominantly associated with nucleotide metabolism and the generation of precursor metabolites and energy. Cellular components (CC) and molecular functions (MF) were highly enriched in oxidoreductase complexes and electron transfer/active transmembrane transporter activities, respectively (Figure 1D). Consistent with these functional annotations, KEGG pathway analysis highlighted oxidative phosphorylation as a primary mechanism targeted by octanoic acid (Figure 1E).

For decanoic acid, GO analysis indicated a strong association with fatty acid and cellular ketone metabolic processes, as well as responses to oxygen levels. Enriched cellular components included secretory granule and vesicle lumens, whereas molecular functions were linked to organic and carboxylic acid binding (Figure 2D). Further KEGG pathway analysis highlighted the PPAR signaling pathway and the cAMP signaling pathway as the crucial mechanisms underlying the anti‐T2DM effects of decanoic acid (Figure 2E).

### 3.2 Molecular Docking Results

To validate the network pharmacology predictions, the key hub targets PPARA, PPARG, ICAM1, and CXCL8 were selected as receptors for molecular docking with octanoic acid and decanoic acid. Binding affinity was evaluated based on the lowest binding energy, where values below 0 kcal/mol indicate spontaneous binding conformations (Trott and Olson 2010).

**FIGURE 3:**
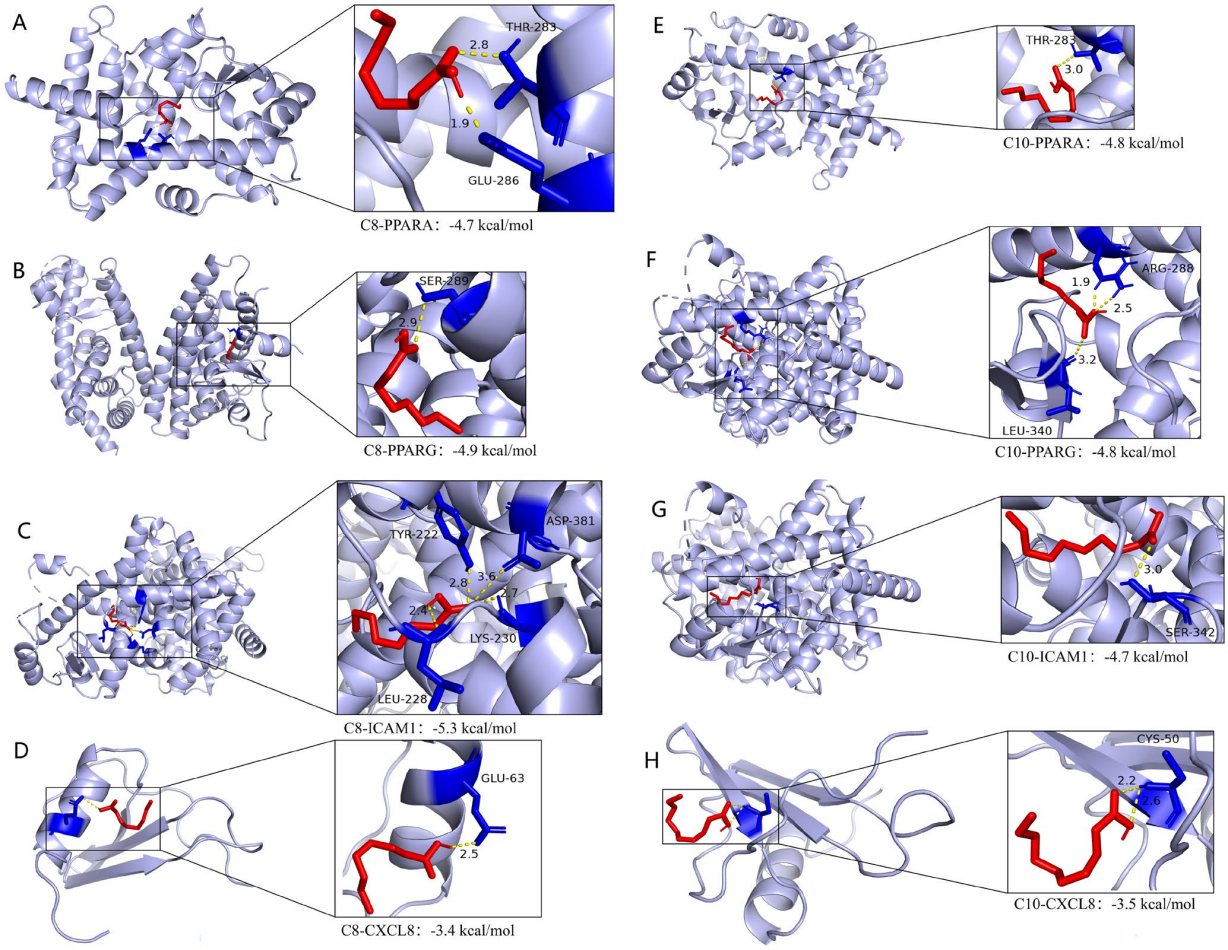
Molecular docking results of octanoic acid (C8) and decanoic acid (C10) with key T2DM targets. (A) octanoic acid‐peroxisome proliferator‐activated receptor alpha (PPARA); (B) octanoic acid‐peroxisome proliferator‐activated receptor gamma (PPARG); (C) octanoic acid‐intercellular adhesion molecule 1 (ICAM1); (D) octanoic acid‐C‐X‐C motif chemokine ligand 8 (CXCL8); (E) decanoic acid‐PPARA; (F) decanoic acid‐PPARG; (G) decanoic acid‐ICAM1; (H) decanoic acid‐CXCL8. The structural models illustrate the binding conformations and interactions between the ligands (octanoic acid, decanoic acid) and the therapeutic targets, further supporting their potential modulatory effects in T2DM pathogenesis.

For octanoic acid, the calculated binding energies with PPARA, PPARG, ICAM1, and CXCL8 were −4.7, −4.9, −5.3, and −3.4 kcal/mol, respectively (Figure 3A–D). Similarly, decanoic acid demonstrated comparable binding affinities, yielding binding energies of −4.8, −4.8, −4.7, and −3.5 kcal/mol with the same respective targets (Figure 3E–H). These results confirm that both medium‐chain fatty acids can successfully dock into the active pockets of these core T2DM‐related targets.

### 3.3 Histopathological Assessment of Renal Injury and Fibrosis

**FIGURE 4:**
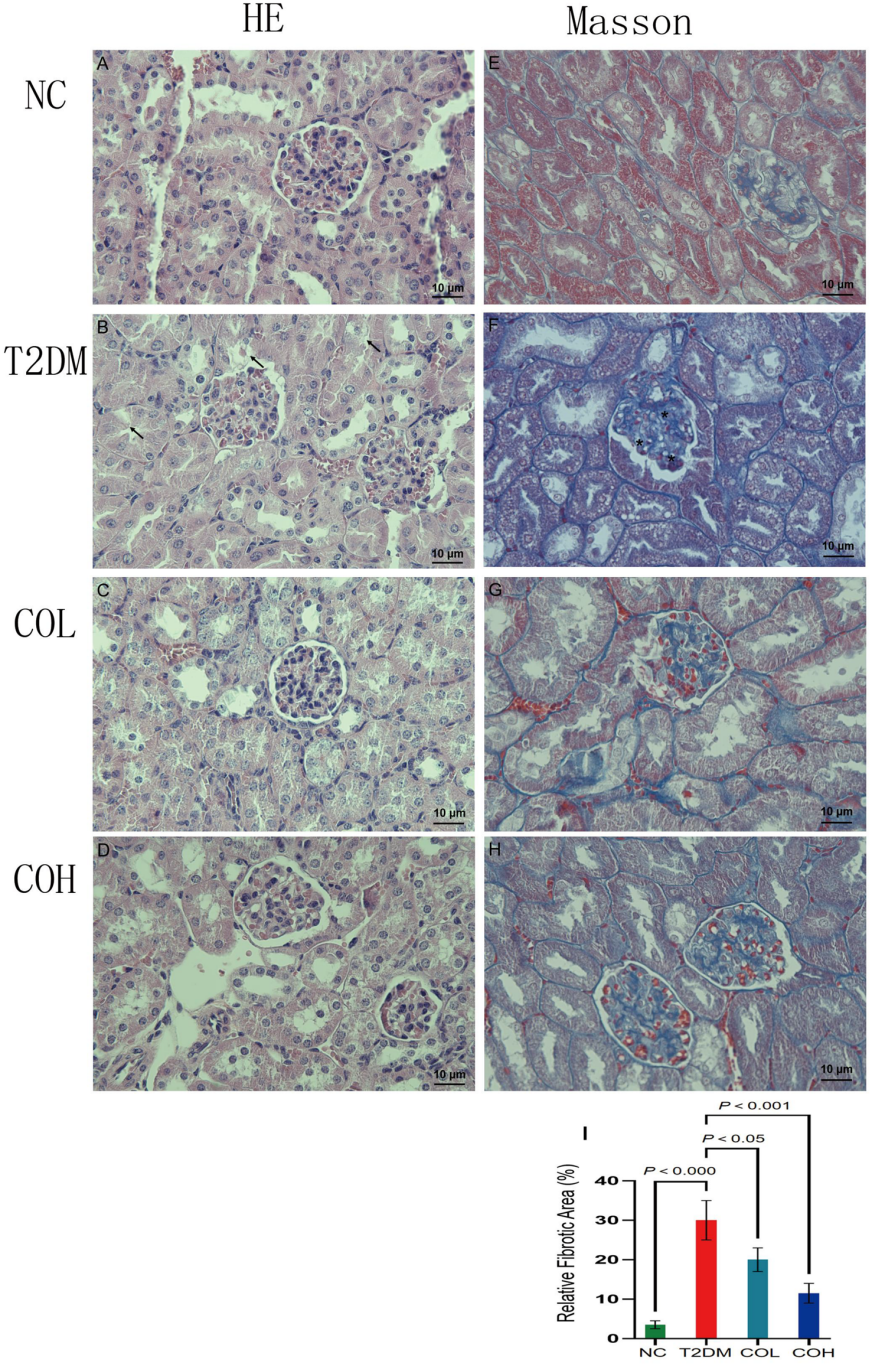
Histopathological evaluation of renal tissues by H&E and Masson's trichrome staining in experimental mice. (A–D) Representative H&E staining images of the NC, T2DM, low‐dose virgin coconut oil (COL), and high‐dose virgin coconut oil (COH) groups. Black arrows in (B) indicate structural abnormalities such as inflammatory infiltration and glomerular damage. (E–H) Representative Masson's trichrome staining images of the NC, T2DM, COL, and COH groups. Black asterisks (*) in (F) highlight areas of severe collagen deposition and fibrosis. Scale bars = 10 μm. (I) Quantitative bar chart of the relative fibrotic area (%) calculated from Masson's trichrome staining. Data are expressed as mean ± SD (*n* = 3 per group). Statistical significance was determined using a one‐way analysis of variance (ANOVA) followed by Tukey's post hoc test.

Histopathological evaluation by H&E staining revealed intact glomerular structures in the normal control (NC) group, characterized by uniformly distributed capillary loops without congestion or atrophy, and tightly aligned tubular epithelial cells devoid of vacuolar degeneration, detachment, or inflammatory cell infiltration. In contrast, the T2DM group exhibited thickened glomerular basement membranes, partial capillary loop collapse, vacuolar degeneration of tubular epithelial cells, and significant interstitial inflammatory infiltration. Following treatment, both the low‐dose (COL) and high‐dose (COH) CO groups demonstrated improved glomerular capillary architecture, reduced tubular vacuolization, and diminished inflammatory cell infiltration compared to the untreated T2DM group (Figure 4A–D).

To assess renal fibrosis, Masson's trichrome staining was utilized to visualize collagen fibers (stained blue). The NC group displayed minimal perivascular collagen deposition with scarce fibrotic lesions in the glomeruli. Conversely, the T2DM group showed extensive interstitial collagen accumulation, reflecting severe fibrosis. In the COL and COH treatment groups, peritubular fibrosis and interstitial collagen deposition were visibly reduced compared to the T2DM group (Figure 4E–I).

### 3.4 Effects of CO on Metabolic, Biochemical, and Molecular Parameters in T2DM Mice

**FIGURE 5:**
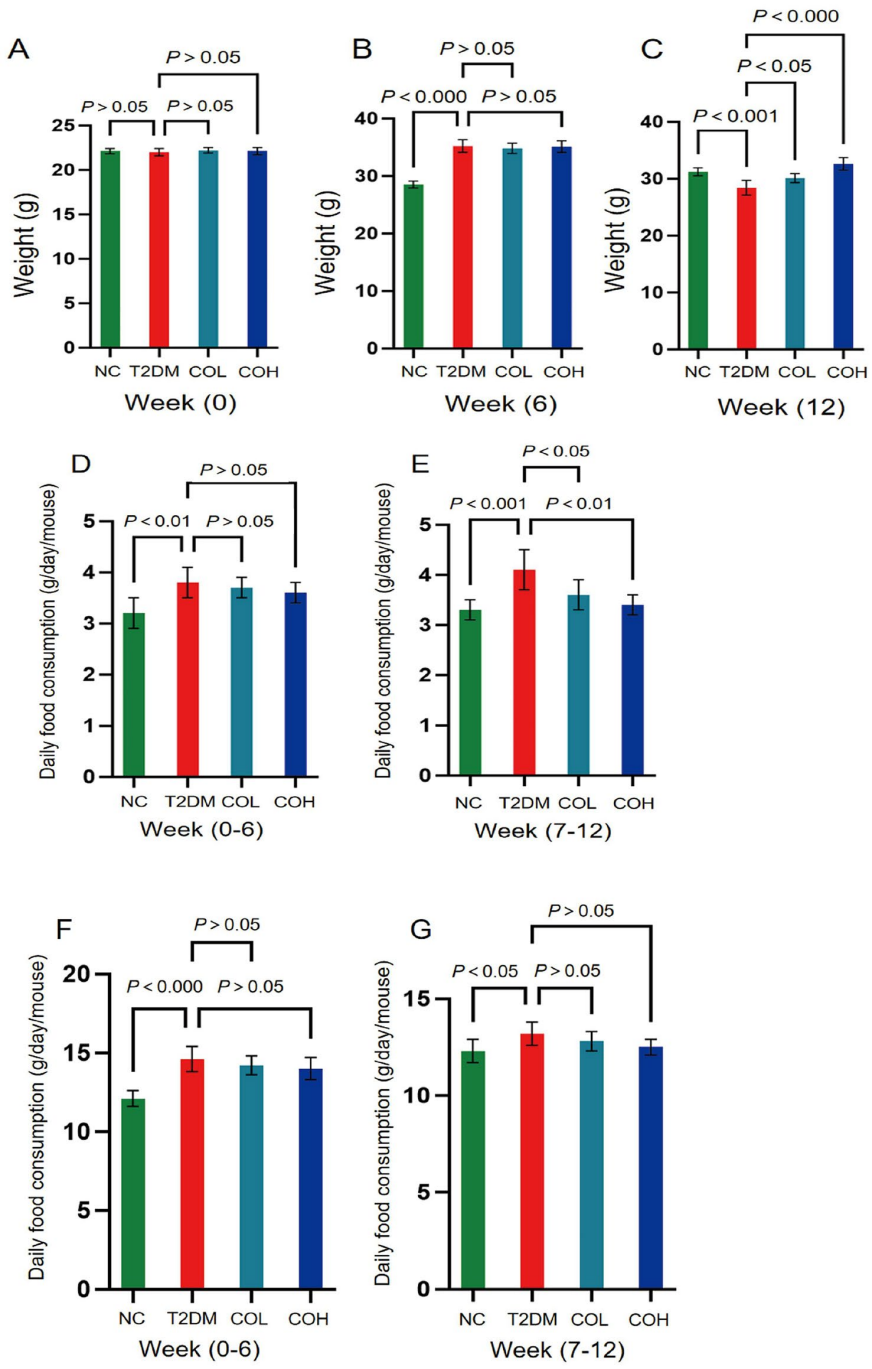
Body weight, dietary intake, and energy consumption in mice across NC, T2DM, COL, and COH groups. (A) Initial body weight at baseline; (B) Body weight at week 6; (C) Body weight at week 12; (D) Daily food intake during weeks 0–6; (E) Daily food intake during weeks 7–12; (F) Daily energy intake during weeks 0–6; (G) Daily energy intake during weeks 7–12. Data are expressed as mean ± SD (*n* = 6 per group). Statistical significance was determined using one‐way ANOVA followed by Tukey's post hoc test.

At the initiation of the experiment, baseline body weights were comparable across all groups (*p* > 0.05) (Figure 5A). After 6 weeks, the T2DM group exhibited significantly higher body weights compared to the NC group (*p* < 0.05), with no significant differences observed in the COL and COH treatment groups relative to the T2DM group (*p* > 0.05) (Figure 5B). However, by week 12, the T2DM group displayed marked weight loss compared to the NC group (*p* < 0.05). Treatment with either low or high doses of CO significantly attenuated this weight loss, maintaining body weights notably higher than those of the untreated T2DM mice (*p* < 0.05) (Figure 5C).

Analysis of dietary patterns during the first 6 weeks revealed that the T2DM group consumed significantly more food and energy than the NC group (*p* < 0.05); however, CO treatment did not significantly alter these parameters relative to the T2DM group during this phase (*p* > 0.05) (Figure 5D,F). In the subsequent phase (weeks 7–12), the T2DM group maintained elevated food and energy intake. Notably, the COL and COH groups exhibited significantly reduced average daily food consumption compared to the T2DM group (*p* < 0.05), although energy intake remained statistically unchanged (*p* > 0.05) (Figure 5E,G).

**FIGURE 6:**
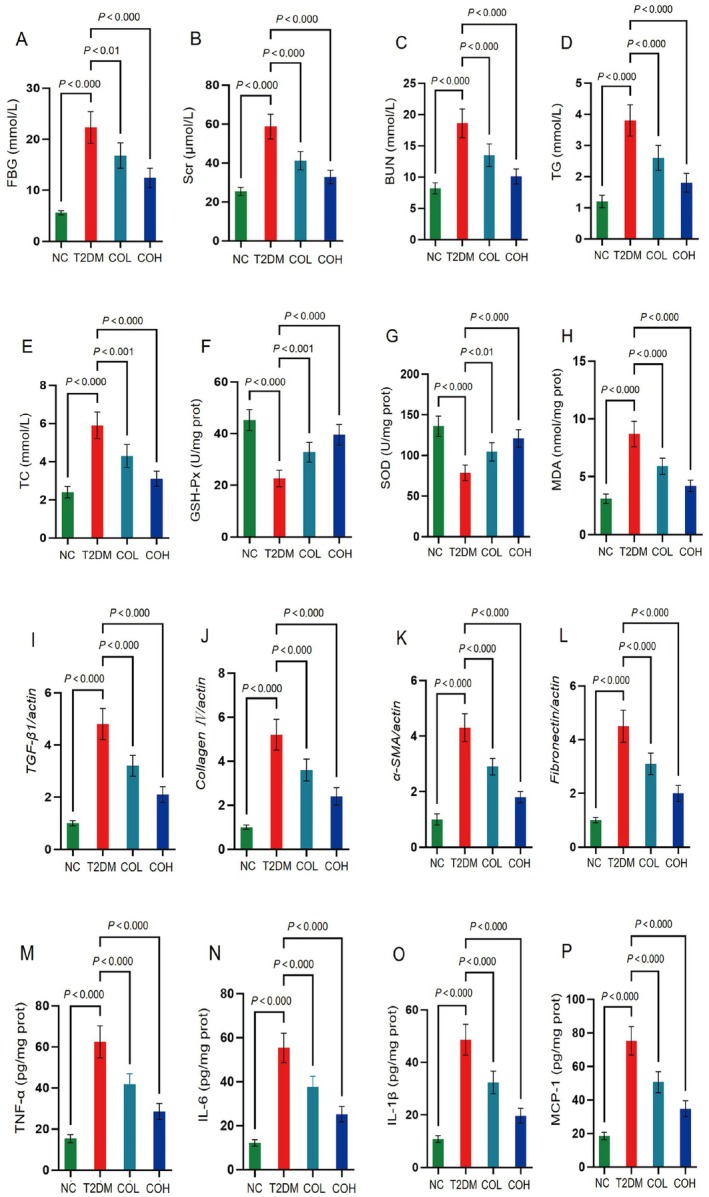
Serum biochemical parameters, renal inflammatory factors, and oxidative stress‐related markers in mice. (A) Fasting blood glucose (FBG); (B) Serum creatinine (Scr); (C) Blood urea nitrogen (BUN); (D) Triglycerides (TG); (E) Total cholesterol (TC); (F) Glutathione peroxidase (GSH‐Px); (G) Superoxide dismutase (SOD); (H) Malondialdehyde (MDA); (I) Transforming growth factor beta 1 (TGF‐β1) mRNA expression; (J) Collagen IV mRNA expression; (K) Alpha‐smooth muscle Actin (α‐SMA) mRNA expression; (L) Fibronectin mRNA expression; (M) Tumor necrosis factor alpha (TNF‐α) mRNA expression; (N) Interleukin 6 (IL‐6) mRNA expression; (O) Interleukin 1 beta (IL‐1β) mRNA expression; (P) Monocyte chemoattractant protein‐1 (MCP‐1) mRNA expression. Data are presented as mean ± SD (*n* = 6 per group). Experimental groups include NC, T2DM, COL, and COH. Statistical significance was determined using one‐way ANOVA followed by Tukey's post hoc test.

Biochemical analysis showed that CO treatment significantly decreased serum levels of FBG, Scr, BUN, TG, and TC in T2DM mice compared to the untreated diabetic group (*p* < 0.05) (Figure 6A–E). In renal tissues, CO administration elevated the concentrations of the antioxidant enzymes GSH‐Px and SOD, while simultaneously reducing MDA levels (*p* < 0.05) (Figure 6F–H). Furthermore, CO treatment significantly suppressed the mRNA expression of key inflammatory mediators, including TNF‐α, IL‐6, IL‐1β, and MCP‐1 (*p* < 0.05) (Figure 6M–P). Similarly, the expression of critical fibrotic markers—TGF‐β, Collagen IV, α‐SMA, and Fibronectin—was significantly downregulated in the kidneys of CO‐treated diabetic mice (*p* < 0.05) (Figure 6I–L).

### 3.5 Effects of CO on Gut Microbiota Composition in T2DM Mice

Given the observed improvements in metabolic and renal parameters—particularly in the high‐dose CO group (COH)—we next investigated the effects of CO on the gut microbiota and serum metabolome of T2DM mice.

**FIGURE 7:**
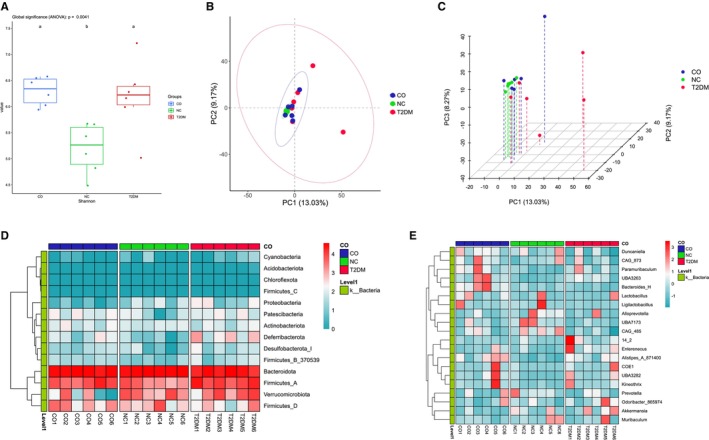
Gut microbiota 16S rRNA sequencing analysis results in mice. (A) α‐diversity (Shannon Index); (B) β‐diversity evaluated by 2D Principal Coordinate Analysis (PCoA); (C) β‐diversity evaluated by 3D PCoA; (D) Phylum‐level gut microbial heatmap; (E) Genus‐level gut microbial heatmap. Experimental groups (*n* = 6 per group) include NC, T2DM, and coconut oil (CO).

Analysis of α‐diversity revealed no significant differences in Shannon indices among the NC, T2DM, and COH groups (Figure 7A). However, PCoA for β‐diversity demonstrated a clear structural separation between the microbiota of the NC and T2DM groups. Furthermore, the COH group clustered distinctly away from the untreated T2DM group, indicating a significant shift in microbial community structure following CO intervention (Figure 7B,C).

At the phylum level, the T2DM group displayed significantly increased relative abundances of Deferribacteres and Firmicutes_A, along with reduced Firmicutes_D compared to the NC group. Treatment with CO notably reduced the abundances of Deferribacteres, Verrucomicrobia, and Proteobacteria relative to the T2DM group (Figure 7D).

At the genus level, the T2DM group exhibited elevated relative abundances of *Paramuribaculum*, *Enterenecus*, *Kineothrix*, *Odoribacter_865974*, *Muribaculum*, *Alistipes*, and *Akkermansia*, alongside decreased *Ligilactobacillus* and *Prevotella* compared to the NC group. Conversely, CO treatment significantly reduced the abundances of *Enterenecus* and *Odoribacter* while increasing the proportions of *Bacteroides_H*, *Lactobacillus*, and *CAG_485* (Figure 7E).

### 3.6 Effects of CO on the Serum Metabolome in T2DM Mice

**FIGURE 8:**
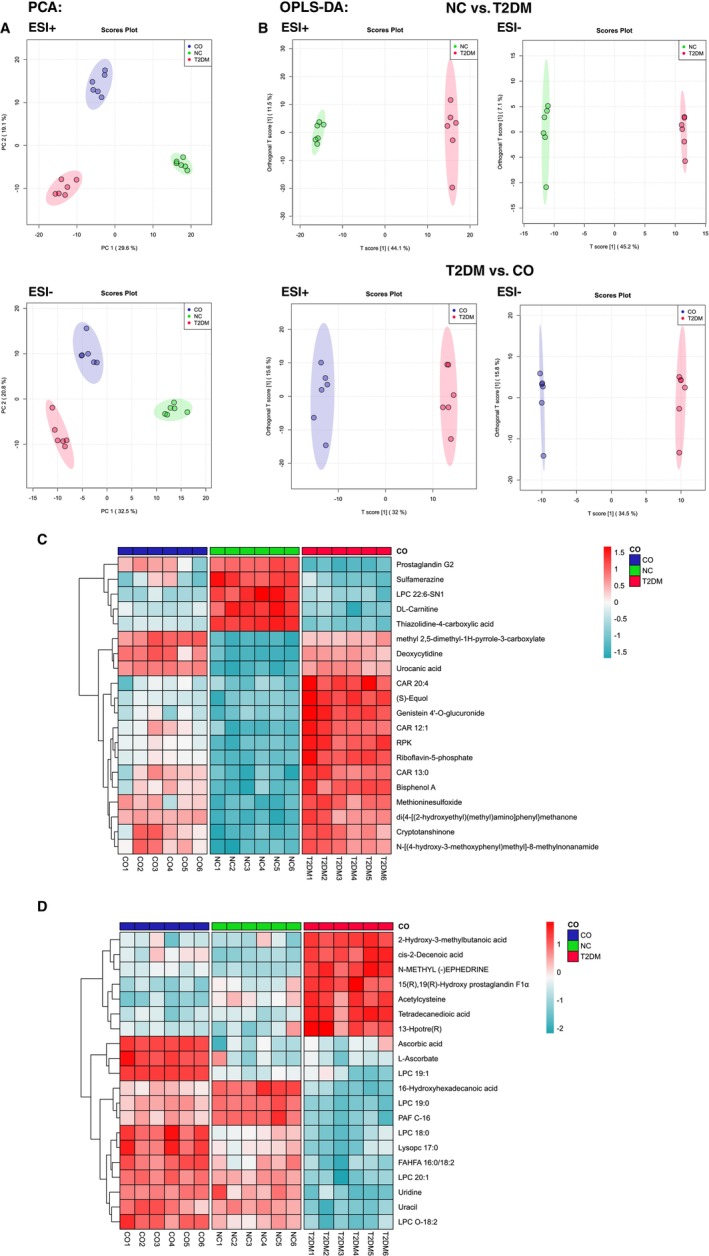
Untargeted serum metabolomics analysis in mice. (A) Principal component analysis (PCA) plots in positive electrospray ionization (ESI+) and negative electrospray ionization (ESI−) modes; (B) Orthogonal partial least squares‐discriminant analysis (OPLS‐DA) plots in ESI+ and ESI− modes; (C) Heatmap of differential serum metabolites in ESI+ mode; (D) Heatmap of differential serum metabolites in ESI− mode. Significant differential metabolites were identified based on a variable importance in projection (VIP) threshold > 1.0 and a two‐tailed *t*‐test (*p* < 0.05). Analyses were performed on normal control (NC), type 2 diabetes mellitus (T2DM), and CO groups (*n* = 6 per group).

Principal component analysis (PCA) of the untargeted serum metabolomics data revealed distinct clustering and clear separation among the NC, T2DM, and COH groups in both positive (ESI+) and negative (ESI−) ion modes (Figure 8A). Subsequent OPLS‐DA was performed to maximize group separation and identify differential metabolites, utilizing a VIP score > 1 and *p* < 0.05 as the selection criteria (Figure 8B).

**FIGURE 9:**
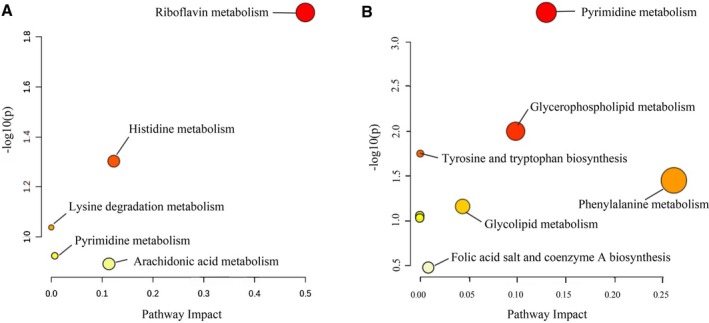
CO‐regulated serum metabolic pathways in T2DM mice. (A) ESI+ model; (B) ESI− model. Pathway enrichment analysis was performed based on untargeted serum metabolomics profiling (*n* = 6 per group).

Hierarchical clustering, visualized via heatmaps, illustrated that CO treatment significantly altered the relative abundances of various differential metabolites in T2DM mice (Figure 8C,D). These structurally identified compounds included metabolites associated with inflammation (e.g., prostaglandin G2, 15(R),19(R)‐hydroxy prostaglandin F1a, N‐methyl (−)ephedrine), vitamin synthesis (e.g., sulfamerazine, RPK, riboflavin‐5‐phosphate, ascorbic acid), and lipid metabolism (e.g., tetradecanedioic acid, LPC 19:1, LPC 19:0, LPC 20:1, LPC 18:0). Pathway enrichment analysis further mapped these altered metabolites to several key biochemical pathways, primarily including riboflavin metabolism, arachidonic acid metabolism, pyrimidine metabolism (Figure 9).

## 4 Discussion

DKD is a common microvascular complication in T2DM patients, with pathogenesis involving chronic inflammation, oxidative stress, and renal fibrosis. In recent years, the nephroprotective effects of natural products have attracted considerable attention (Cao et al. 2022; Josa et al. 2024). CO, rich in MCFAs (primarily octanoic acid and decanoic acid), exhibits notable antioxidant and anti‐inflammatory properties, suggesting potential therapeutic benefits in DKD (Cao et al. 2022; Gao and Chen 2022; Raillon et al. 2025). This study combined network pharmacology, animal experiments, and multi‐omics analyses to investigate the mechanisms underlying CO‐mediated amelioration of renal injury in T2DM.

The CO used in this study contained > 57% octanoic acid and > 42% decanoic acid, making it an ideal model for studying MCFAs' biological effects. While previous studies have demonstrated the anti‐inflammatory and antioxidant properties of MCFAs, systematic investigations into their therapeutic potential for DKD remain scarce. To elucidate the role of high‐purity MCFAs (octanoic acid + decanoic acid) in DKD management, we first employed network pharmacology, revealing that octanoic acid and decanoic acid modulate multiple inflammation‐related factors, including ICAM1, PPARA, PPARG, CRP, IL‐1β, and CXCL8. KEGG pathway analysis further indicated associations with DKD‐relevant pathways, such as neurodegeneration‐multiple diseases, prion disease, oxidative phosphorylation, PPAR signaling, neuroactive ligand‐receptor interaction, and cAMP signaling.

Molecular docking simulations demonstrated stable binding between octanoic acid/decanoic acid and pro‐inflammatory targets (PPARA, PPARG, ICAM1, CXCL8). These findings align with existing literature; for instance, PPARα/γ are established targets of MCFAs, with octanoic acid shown to activate PPARα signaling and suppress pro‐inflammatory cytokine release in high‐fat diet models. CXCL8 (IL‐8) and ICAM1 are direct downstream effectors of the NF‐κB pathway, pivotal in DKD progression (Jung and Moon 2021). Docking results suggest that octanoic acid/decanoic acid may inhibit these proteins through steric hindrance or conformational changes, thereby attenuating inflammatory signaling—a mechanism consistent with recent reports of MCFAs suppressing TLR4/NF‐κB in colitis (Chen et al. 2021; Peña‐Vázquez et al. 2025). Crucially, while network pharmacology and molecular docking pinpointed the direct host‐side receptors (such as PPARα/γ and key inflammatory cytokines) for CO's active components, DKD is a systemic disease driven by complex organ crosstalk. Therefore, we further hypothesized that these direct host‐receptor interactions are intimately linked with broader systemic shifts. To fully elucidate this, we combined these computational predictions with in vivo multi‐omics analyses to map a comprehensive “gut microbiota‐metabolism‐inflammation” axis.

Animal experiments confirmed that oral CO significantly reduced renal expression of TNF‐α, IL‐6, IL‐1β, and MCP‐1 in T2DM mice. This effect likely involves not only NF‐κB inhibition but also JAK/STAT modulation (Haftcheshmeh et al. 2022). IL‐6 drives renal fibrosis via JAK1/STAT3, supported by the downregulation of α‐SMA and fibronectin observed here (Ji et al. 2025; Liu et al. 2023). Similarly, TGF‐β/Smad suppression may contribute to octanoic acid/decanoic acid‐mediated antifibrotic effects, as reported in obese mice (Hu et al. 2021; Xu et al. 2025).

Renal antioxidant assays revealed elevated GSH‐Px and SOD activity, alongside reduced MDA levels in CO‐treated mice, implicating Nrf2/ARE activation (Gao et al. 2021). Prior studies indicate that decanoic acid enhances antioxidant enzyme expression via Keap1‐Nrf2 interactions, a pathway corroborated here (Chae et al. 2023; Dunn et al. 2023). Additionally, rapid β‐oxidation of MCFAs may mitigate mitochondrial ROS production, indirectly alleviating oxidative stress (Guerra et al. 2022; Mett 2021).

CO treatment also improved metabolic parameters (e.g., fasting glucose), renal function, and inflammatory/oxidative markers. Notably, CO attenuated glomerular injury and interstitial fibrosis, accompanied by downregulated TGF‐β and collagen I expression, confirming the nephroprotective role of high‐purity MCFAs in diabetic renal injury (Mohany et al. 2021; Ram et al. 2022).

Mechanistic studies suggested that MCFAs may act through the gut microbiota‐host metabolism axis (Liang et al. 2023; Zhang, Yu, et al. 2023). 16S rRNA sequencing revealed CO‐induced shifts in microbial composition, including suppression of pro‐inflammatory *Enterococcus* and *Odoribacter*, while enriching beneficial *Bacteroides_H* and *Lactobacillus*. *Enterococcus* exacerbates inflammation via NF‐κB activation, correlating with DKD progression (Chen et al. 2023; Zheng et al. 2024). *Odoribacter* generates secondary bile acids (e.g., deoxycholic acid), which impair gut barrier function, permitting LPS translocation and systemic inflammation via TLR4/NF‐κB (Sato et al. 2021; Shi et al. 2023; Sun et al. 2021). Thus, CO's modulation of these taxa may directly ameliorate T2DM pathology.

Elevated *Bacteroides_H* (a short‐chain fatty acid (SCFA) producer) and *Lactobacillus* further support CO's benefits (He et al. 2025). SCFAs (acetate, propionate) suppress NLRP3 inflammasome activation (via GPR43) and enhance gut barrier integrity (via HDAC inhibition), reducing endotoxemia (Fang et al. 2024; Farhadipour et al. 2023; Kopczyńska and Kowalczyk 2024). Lactate from *Lactobacillus* acidifies the gut lumen, inhibiting pathogenic *Enterococcus* (Zeise et al. 2021). Subsequent studies will employ antibiotic depletion (e.g., vancomycin for *Lactobacillus*) to validate these mechanisms (Isaac et al. 2022; Rubin et al. 2022).

Our untargeted metabolomic analysis revealed that oral administration of CO substantially altered the serum metabolic profile in T2DM mice, with these metabolic perturbations potentially conferring indirect nephroprotective effects through mechanisms including gut barrier enhancement and systemic inflammation mitigation. Notably, CO treatment induced a marked elevation in serum prostaglandin G2 (PGG2) levels. Mechanistic explorations suggest two potential pathways through which MCFAs in CO may modulate PGG2 biosynthesis: First, hepatic metabolization of MCFAs into ketone bodies likely activates the PPARγ signaling cascade, subsequently upregulating cyclooxygenase‐1 (COX‐1) activity to promote arachidonic acid conversion into PGG2 (Tsai et al. 2011; van Zadelhoff and van der Stelt 2023). Second, MCFAs‐mediated suppression of fatty acid β‐oxidation may preserve arachidonic acid pools for prostaglandin synthetic pathways (Angelini et al. 2021; Zeng and Zhang 2025; Zhang et al. 2024). Importantly, the dynamic fluctuation of PGG2 concentrations likely reflects CO's global modulation of the arachidonic acid metabolic network rather than exhibiting simplistic pro−/anti‐inflammatory properties (Wang et al. 2021). Future investigations employing targeted lipidomics will systematically quantify downstream metabolites including prostaglandin E2 (PGE2) and prostaglandin D2 (PGD2) to fully delineate PGG2's functional significance within this metabolic regulatory landscape.

Non‐targeted metabolomics revealed a significant elevation in sulfamerazine, a sulfonamide antibiotic potentially associated with microbial toluene metabolism pathways, within the serum of CO‐treated T2DM mice. Given that T2DM pathogenesis typically involves gut microbiota dysbiosis (characterized by altered Firmicutes/Bacteroidetes ratios), we hypothesize that the medium‐chain triglycerides (octanoic acid and decanoic acid components) in CO may selectively promote the proliferation of beneficial microbiota such as *Lactobacillus* and *Bacteroides* species. This microbial remodeling likely upregulates folate/vitamin K‐dependent metabolic cascades, subsequently leading to sulfonamide biotransformation products accumulation and consequent sulfamerazine concentration elevation in systemic circulation (Ovung and Bhattacharyya 2021). Notably, this observation suggests a previously unrecognized interaction between dietary lipids, gut microbiota metabolic networks, and xenobiotic processing pathways in diabetic conditions.

Following oral CO administration, T2DM mice exhibited significant reductions in serum levels of carnitine‐conjugated fatty acids (CARs), including arachidonoylcarnitine (CAR 20:4), dodecenoylcarnitine (CAR 12:1), and tridecanoylcarnitine (CAR 13:0), suggesting enhanced fatty acid oxidation and improved insulin sensitivity (Tamas et al. 2024; Weiss et al. 2023). CARs, the obligatory transporters facilitating long−/medium‐chain fatty acid entry into mitochondria for β‐oxidation, typically accumulate during impaired fatty acid catabolism (Angelini et al. 2021). Under diabetic insulin resistance, diminished mitochondrial oxidative capacity elevates systemic CAR concentrations. Mechanistically, CO alleviates this metabolic perturbation through multi‐level interventions: First, MCFAs in CO bypass the carnitine shuttle system for direct mitochondrial import, thereby decreasing conjugation demand for long‐chain species like CAR 20:4 (Kanta et al. 2025). Second, CO‐derived octanoic acid and decanoic acid generate acetyl‐CoA surplus that stimulates TCA cycle flux, simultaneously reducing free fatty acid accumulation and subsequent generation of medium/short‐chain acylcarnitines (CAR 12:1, CAR 13:0) (Felix et al. 2021). Third, CO's anti‐inflammatory and insulin‐sensitizing properties mitigate adipose tissue lipolysis, limiting free fatty acid mobilization to the liver—the primary CAR biosynthesis site (Grabner et al. 2021). These concerted actions, potentially mediated through PPARα activation, collectively rewire fatty acid trafficking patterns and downregulate CAR production in the diabetic state.

The estrogen‐like isoflavonoid metabolite (S)‐equol, a bacterial biotransformation product of daidzein generated by specific gut microbiota (e.g., *Slackia equolifaciens* , *Adlercreutzia equolifaciens* ), exhibited reduced serum concentrations in CO‐treated mice (Gong et al. 2023). Since all groups received identical dietary formulations, alterations in circulating equol levels primarily reflect shifts in gut microbial composition and metabolic activity. We propose that MCFAs in CO may selectively suppress the proliferation of equol‐producing bacterial taxa, thereby attenuating systemic (S)‐equol exposure.

Retinol‐binding protein 4 (RBP4), predominantly secreted by hepatocytes and adipocytes, facilitates systemic retinol (vitamin A's bioactive form) trafficking from hepatic stores to peripheral tissues, with its circulating levels correlating with insulin resistance severity (Fan and Hu 2024). Elevated serum RBP4 exacerbates metabolic dysfunction by activating proinflammatory JNK/NF‐κB signaling, mechanistically linking it to obesity, type 2 diabetes mellitus (T2DM), and metabolic syndrome pathogenesis (Yang et al. 2022). The observed reduction of RBP4 in CO‐treated diabetic mice aligns with CO's established insulin‐sensitizing and anti‐inflammatory properties. Riboflavin‐5′‐phosphate (FMN), the bioactive coenzyme form of vitamin B_2_ and precursor to flavin adenine dinucleotide (FAD), functions as an endogenous antioxidant and β‐oxidation facilitator (Yu et al. 2022). CO administration decreased serum FMN concentrations in T2DM mice, potentially attributable to improved mitochondrial efficiency reducing FMN turnover. Furthermore, MCFAs in CO serve as direct energy substrates, diminishing reliance on FMN‐dependent electron transport chain activity (particularly as Complex I's essential cofactor), thereby lowering systemic FMN requirements and subsequent circulating levels (Ruchala et al. 2025).

2‐Hydroxy‐3‐methylbutanoic acid (2‐H3MB), a branched‐chain organic acid generated by gut microbiota (e.g., Clostridium, Bacteroides) during branched‐chain amino acid (BCAA) metabolism, exhibits elevated serum concentrations in metabolic dysregulation linked to obesity and T2DM (Sun et al. 2025). In diabetic conditions, impaired skeletal muscle BCAA uptake contributes to systemic 2‐H3MB accumulation, while insulin resistance exacerbates this by promoting proteolysis and subsequent BCAA release (De Bandt et al. 2022). The reduction of 2‐H3MB in CO‐treated diabetic mice aligns with CO's potential to ameliorate gut dysbiosis and restore BCAA metabolic homeostasis, though this hypothesis warrants further experimental validation.

N‐Methyl(−)ephedrine (NME), a sympathomimetic amine structurally analogous to epinephrine, is a bioactive microbial metabolite derived from dietary precursors by certain gut bacteria (Beyoğlu et al. 2022). This compound enhances hepatic gluconeogenesis and lipolysis while exacerbating insulin resistance (Dodonova et al. 2023). In T2DM mice, gut dysbiosis may promote NME biosynthesis, whereas CO‐mediated restoration of microbial homeostasis correlates with reduced systemic NME levels.

15(R),19(R)‐Hydroxy prostaglandin F1α (PGF1α), an arachidonic acid (AA) metabolite, exhibits pro‐inflammatory properties and contributes to insulin resistance development (Xie et al. 2024). The MCFAs in CO attenuate PGF1α biosynthesis through dual mechanisms: (1) suppression of the NF‐κB/COX‐2 signaling axis via their anti‐inflammatory activity (Richter et al. 2023), and (2) enhancement of insulin sensitivity coupled with inhibition of hormone‐sensitive lipase in adipose tissue, thereby reducing free fatty acid release (Lundsgaard et al. 2021). This metabolic modulation decreases phospholipase A2 activation and subsequent AA availability, ultimately diminishing PGF1α synthesis (Tsai et al. 2022).

Tetradecanedioic acid, a dicarboxylic acid metabolite, is generated through ω‐oxidation of long‐chain fatty acids such as myristic acid (C14:0). The elevation of tetradecanedioic acid and related dicarboxylates in T2DM mice reflects impaired fatty acid metabolism and compromised mitochondrial β‐oxidation (Strittmatter et al. 2022). CO‐derived MCFAs bypass these metabolic constraints by directly entering mitochondrial β‐oxidation, thereby reducing the accumulation of long‐chain fatty acids (e.g., C14:0) and decreasing reliance on ω‐oxidation (Panov et al. 2022). This shift attenuates tetradecanedioic acid production, suggesting a concomitant reduction in systemic oxidative stress in T2DM mice.

Following oral CO administration, we observed a significant increase in serum levels of ascorbic acid and other antioxidative metabolites in T2DM mice, indicating marked attenuation of systemic inflammation and oxidative stress. CO‐mediated intestinal anti‐inflammatory effects were accompanied by upregulated expression of sodium‐dependent vitamin C transporters, collectively enhancing intestinal ascorbic acid absorption and elevating its circulating concentrations (Woubshete et al. 2024). Furthermore, CO may improve ascorbic acid recirculation by ameliorating hepatic steatosis in T2DM mice, thereby contributing to increased serum ascorbate levels (Islam et al. 2021). These findings demonstrate CO's capacity to potently enhance antioxidative and anti‐inflammatory defenses in T2DM, which likely underlies its therapeutic benefits in mitigating diabetic symptoms and preventing renal injury progression.

Untargeted metabolomics revealed elevated levels of multiple lysophosphatidylcholines (LPCs, including LPC 19:1, LPC 19:0, LPC 20:1, LPC 18:0, LPC O‐18:2, and LysoPC 17:0). LPCs exhibit dualistic bioactivity, displaying both pro‐inflammatory and anti‐inflammatory properties (Casati et al. 2021). Acute exposure facilitates pro‐inflammatory responses via TLR4/NF‐κB pathway activation, whereas prolonged exposure leads to hydrolytic conversion into anti‐inflammatory lysophosphatidic acid (LPA) by autotaxin (ATX)/lysophospholipase D (Mori et al. 2007). We hypothesize that MCFAs in CO suppress systemic inflammation, thereby downregulating ATX activity and decelerating LPA metabolism, ultimately inducing serum LPC accumulation. However, the precise mechanistic interplay warrants further targeted lipidomics investigation, including quantitative profiling of hepatic and systemic LPC/PC ratios and LPA concentrations.

This study has several limitations that warrant acknowledgment. First, while our in vivo multi‐omics and computational analyses delineate a systemic “gut microbiota‐metabolism” axis, the lack of in vitro cellular models and specific pharmacological inhibitors (e.g., target gene silencing or PPAR antagonists) limits our ability to definitively confirm the exact downstream molecular signaling cascades. Consequently, our experimental validation of these specific pathways was primarily limited to downstream transcriptional changes (e.g., qRT‐PCR of *IL‐1β*), and direct protein‐level evaluations using Western blotting or immunohistochemistry will be a priority in our future investigations. Second, this study lacks a standard pharmacological positive control group (e.g., metformin or ACE inhibitors), which would have provided a valuable benchmark to evaluate the relative clinical efficacy of CO against established therapies. Third, although we confirmed the renoprotective effects of CO, its precise absorption and metabolic kinetics remain unresolved. Our experimental design utilized only two concentration gradients, which restricts our ability to fully characterize the precise dose–response relationship and optimal pharmacological kinetics. Furthermore, the causal mechanistic link between CO and gut microbiota modulation requires deeper investigation; future studies will employ fecal microbiota transplantation to delineate the contributory roles of specific bacterial taxa in CO‐mediated renal protection. Finally, given the interspecies variance in MCFA metabolism between rodents and humans, the clinical translatability of these findings warrants further validation. These future investigations, coupled with clinical trials evaluating CO's effects on renal function in diabetic patients, will critically inform the comprehensive assessment of CO's therapeutic potential.

## 5 Conclusion

**FIGURE 10:**
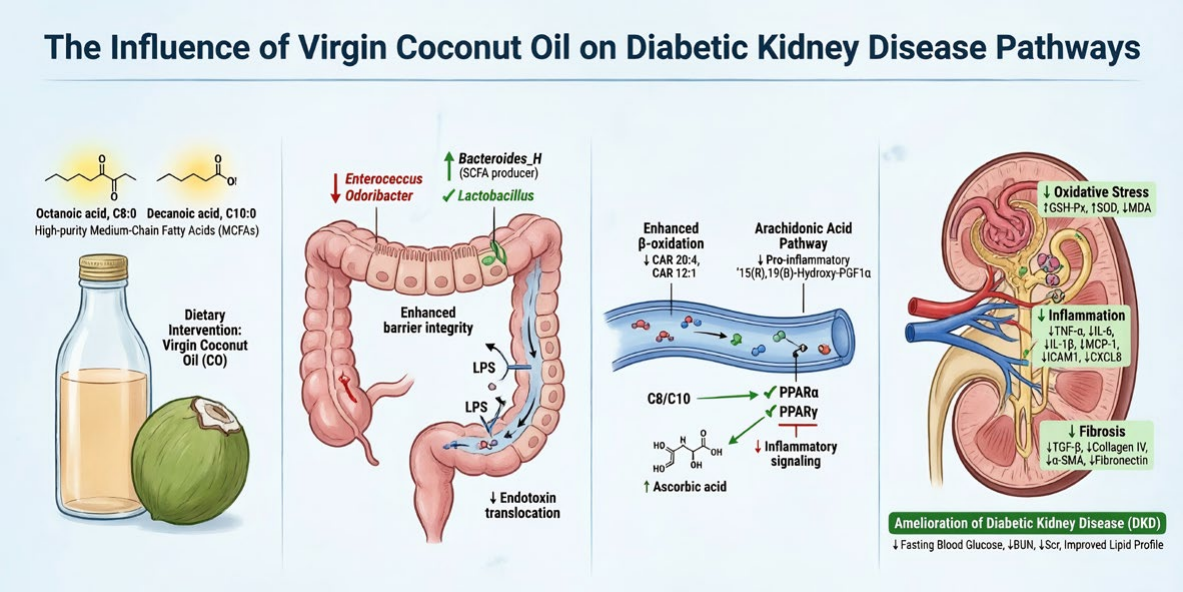
Virgin coconut oil (CO) ameliorates diabetic kidney disease by modulating the “gut microbiota‐metabolism‐inflammation axis” to restore gut barrier integrity, activate PPAR signaling, and subsequently alleviate renal oxidative stress, inflammation, and fibrosis.

This study provides comprehensive mechanistic insights into the nephroprotective potential of CO in T2DM, establishing its multi‐targeted effects on inflammation, oxidative stress, metabolic dysfunction, and gut microbiota homeostasis. Our findings highlight the dynamic interplay between dietary MCFAs, microbial metabolism, and host inflammatory‐oxidative balance in diabetic renal injury. Future investigations employing targeted lipidomics, microbial functional genomics, and clinical validation will be essential to fully delineate CO's therapeutic potential and optimize translational strategies for DKD management. Collectively, this work positions CO as a promising nutraceutical candidate, bridging metabolic regulation and microbiota‐directed interventions to mitigate diabetic nephropathy progression (Figure 10).

## Author Contributions

**Keke Shao:** conceptualization, data curation, investigation, methodology, resources, validation, visualization, writing – original draft. **Yun Cao:** conceptualization, investigation, writing – original draft, methodology, validation, visualization, data curation, resources. **Ruiqi Gao:** conceptualization, data curation, investigation, methodology, resources, validation, visualization. **Xuehui Liu:** conceptualization, data curation, investigation, methodology, visualization, writing – original draft. **Linlin Xu:** data curation, investigation, methodology. **Hao Xu:** conceptualization, investigation, resources, writing – original draft. **Asmaa Ali:** conceptualization, resources, supervision, writing – review and editing. **Xueyun Dong:** conceptualization, data curation, investigation, methodology, visualization, writing – original draft. **Min Chen:** methodology, validation. **Yunhan Xie:** conceptualization, funding acquisition, investigation, resources, writing – original draft. **Liang Wu:** validation, visualization, supervision, writing – review and editing. **Jiayuan He:** methodology, validation. **Pingping Wang:** validation, visualization, supervision, writing – review and editing. **Leilei Zhang:** methodology, validation.

## Funding

This research was funded by Scientific Research Project of Yancheng Municipal Health Commission (Grants YK2024116 and YK2024120).

## Ethics Statement

All animal experiments were carried out with approval by the Ethics Committee of Jiangsu University (protocol code UJS‐IACUC‐AP‐2022032011 and date of approval: January 2022) and adhered to the 3R principles.

## Consent

The authors have nothing to report.

## Conflicts of Interest

The authors declare no conflicts of interest.

## Supporting information


**Table S1:** qPCR primer sequence.

## Data Availability

The data that support the findings of this study are available from the corresponding author upon reasonable request.
