# Supplementary material for: Virgin Coconut Oil Attenuates Diabetic Kidney Disease via Gut Microbiota‐Metabolism‐Inflammation Axis Modulation in Type 2 Diabetic Mice
Source: Food Sci Nutr. 2026 Jun 11;14(6):e71973. doi: 10.1002/fsn3.71973 (PMC13260704; doi:10.1002/fsn3.71973)
Supplement: Supplementary file 1 — Table S1: qPCR primer sequence. [file FSN3-14-e71973-s001.docx]

**Supplementary Materials**

Table S1. qPCR primer sequence

| Genes | Upstream primer（5'→3'） | Downstream primer（5'→3'） |
| --- | --- | --- |
| *TGF-β1* | CTCCCGTGGCTTCTAGTGC | GCCTTAGTTTGGACAGGATCTG |
| *Collagen IV* | GGTGAGACAGGCGAACAAGG | CGGCTGCTTTCATAACTCTC |
| *α-SMA* | GTCCCAGACATCAGGGAGTAA | TCGGATACTTCAGCGTCAGGA |
| *Fibronectin* | CAGTGGGAGACCTCGAGAAG | TCCCTCGGAACATCAGAAAC |
| *TNF-α* | CATCTTCTCAAAATTCGAGTGACAA | TGGGAGTAGACAAGGTACAACCC |
| *IL-6* | TACCACTTCACAAGTCGGAGGC | CTGCAAGTGCATCATCGTTGTTC |
| *IL-1β* | TGCCACCTTTTGACAGTGATG | ATGTGCTGCTGCGAGATTTG |
| *MCP-1* | TTAAAAACCTGGATCGGAACCAA | GCATTAGCTTCAGATTTACGGGT |
| *β-actin* | GGCTGTATTCCCCTCCATCG | CCAGTTGGTAACAATGCCATGT |
